# Early Results of Implementing Rapid Methadone Titration for Hospitalized Patients: A Case Series

**DOI:** 10.1007/s11606-024-09341-1

**Published:** 2025-01-13

**Authors:** Marlene Martin, Taylor Baisey, Sasha Skinner, Leslie Ly, Kristin Slown, Kristin Harter, Oanh Kieu Nguyen, Scott Steiger, Leslie W. Suen

**Affiliations:** 1 University of California, San Francisco, CA USA; 2 San Francisco General Hospital, San Francisco, CA USA

**Keywords:** methadone, fentanyl, rapid methadone, opioid use disorder, hospital, addiction consult

## Abstract

**Objectives:**

With the increase in illicit fentanyl use in the USA, hospitals face challenges managing opioid withdrawal and opioid use disorder (OUD). To improve opioid withdrawal and OUD treatment among hospitalized patients with daily fentanyl use, we developed a rapid methadone titration (RMT) protocol. We describe development, implementation, and outcomes during the first 12 weeks.

**Methods:**

We analyzed electronic health record data of hospitalizations seen by the Addiction Consult Team (ACT) for methadone initiation between 9/11/23 and 12/3/23. Adults aged 18–64 reporting daily fentanyl use, desiring methadone, and without end-stage organ damage or critical illness were RMT eligible. We characterized patients who received RMT, abstracting demographic and clinical characteristics, adverse events graded by the Common Terminology Criteria for Adverse Events (CTCAE), and methadone and additional full agonist opioid (FAO) dosing. Our primary outcome was adverse events. Secondary outcomes included median time to 100 mg of methadone, FAO dosing, and self-directed discharge.

**Results:**

ACT assessed 55 hospitalizations representing 47 patients for RMT eligibility. Among these, 19 (34.5%) hospitalizations representing 17 patients were eligible for and received RMT. Four (21.2%) hospitalizations that received RMT had sedation events, and all were mild or moderate grade by CTCAE. Hospitalizations achieved a median methadone dose of 100 mg by day 6, with FAO doses peaking on day 5. One (5.3%) hospitalization had a self-directed discharge.

**Conclusions:**

With careful patient selection and ACT evaluation, a RMT protocol for hospitalized patients with fentanyl use disorder experienced few adverse events other than mild-moderate sedation, even among those receiving FAO and those with concurrent substance use disorders.

**Supplementary Information:**

The online version contains supplementary material available at 10.1007/s11606-024-09341-1.

## INTRODUCTION

With the rise of illicit fentanyl use in the USA, hospitalizations involving opioid use disorder (OUD) have increased.^1,2^ Fentanyl’s high potency has resulted in challenges with treating OUD and opioid withdrawal during hospitalization. Given the fear of undertreated withdrawal, patients with OUD may delay hospitalization, self-direct their discharge, or use drugs in the hospital.^3–8^ Gold standard treatment with methadone can help alleviate opioid withdrawal during hospitalization, though traditional starting doses are often insufficient to treat withdrawal among individuals using fentanyl.^9^

With the challenges posed by fentanyl, medication initiations for OUD and opioid withdrawal are rapidly evolving. In hospital settings, clinicians have reported using full agonist opioids (FAO) to manage opioid withdrawal and rapid methadone titration (RMT) to more quickly alleviate opioid withdrawal symptoms. The use of RMT during hospitalization is relatively new, and clinicians are actively developing and piloting these among patients with OUD. Prior to RMT, traditional hospital methadone titrations have been guided by outpatient standards developed for patients using less potent opioids such as oxycodone and heroin.^10–12^ These slower titrations were developed with consideration for methadone’s half-life of 24 h, recommending time to allow methadone to reach steady state and reduce the risk of sedation.^13^ However, these methadone titrations may not effectively manage OUD and opioid withdrawal among patients primarily using fentanyl.^9^

To improve opioid withdrawal and OUD treatment among patients initiating methadone, we developed and piloted RMT for hospitalized patients with OUD and daily fentanyl use and present the first 12 weeks of RMT findings. To our knowledge, our RMT protocol is among the fastest currently used. We hope shedding light on its use will help inform larger clinical practice.

## METHODS

### Study Type

To understand RMT implementation and adverse events for this case series, we assessed all hospitalizations involving Addiction Consult Team (ACT) evaluation for methadone initiation between 9/11/23 and 12/3/23.

### Study Setting

We implemented the RMT protocol in an urban, safety-net academic hospital with an electronic health record (EHR) and an interprofessional ACT in San Francisco, where fentanyl is the dominant opioid in the illicit drug supply.^14^

### Study Population and Inclusion and Exclusion Criteria

Those eligible for RMT were adults aged 18–64 self-reporting daily fentanyl use, desiring to initiate methadone, evaluated by ACT, and wanting RMT. The RMT protocol excluded patients with end-stage organ damage or critical illness (e.g., end-stage renal disease, decompensated heart failure, moderate-severe lung disease, end-stage liver disease, QTc > 500, hypoglycemia) by EHR review. We developed the inclusion and exclusion criteria to reduce the risk of oversedation from methadone.

To identify a representative sample of patients newly initiating methadone via the RMT protocol, for the purposes of this case series, we excluded patients who were pregnant, incarcerated, already on methadone as defined by active opioid treatment program enrollment, started on methadone by the primary team ≥ 48 h before the initial ACT visit or started RMT ≥ 48 h after the initial ACT visit, and received RMT but met RMT exclusion criteria. We did review adverse events for patients that met RMT exclusion criteria but received it.

### Developing and Implementing the RMT

Hospital pharmacists, nurses, and addiction medicine physicians developed the RMT protocol via our established Addiction Pharmacy and Therapeutics Subcommittee, which reviews new clinical protocols. The RMT protocol is limited to patients being followed by ACT.

Prior to the RMT protocol, our hospital methadone initiation guideline (herein referred to as traditional) for patients with OUD adapted recommendations from outpatient practices.^10–12,15^ These guidelines aim to reduce the risk of dose-stacking, oversedation, and overdose posed by methadone’s pharmacology, which includes its half-life of 24 h and reaching steady state at 3 to 7 days.^11^ Our traditional methadone guideline recommends initiating methadone at maximum doses of 40 mg on day 1, 50 mg on day 2, and 60 mg on day 3, with up to 10 mg increases every 3 to 5 days thereafter (Table 1). However, this traditional regimen was developed primarily for patients using heroin. Our RMT protocol recommends methadone at up to a maximum of 60 mg on day 1, 80 mg on day 2, 100 mg on day 3, and further evaluation for titration every 3 days thereafter (Table 1). The RMT requires ACT to evaluate patients at peak methadone effect daily before recommending additional doses of methadone. For example, if a patient is getting RMT, they may receive up to 40 mg initially on day 1, with ACT evaluation 2–4 h later to determine if ACT will recommend up to an additional 20 mg more of methadone. Given the required in-person evaluation by ACT, the RMT cannot be implemented when ACT is not present in the hospital (after-hours and Saturdays). We include the detailed RMT protocol and monitoring guidelines in the Appendix.

**Table 1:** Traditional and Rapid Methadone Titration Dosing

Day	Traditional methadone dosing	Rapid methadone dosing
1	Up to 40 mg	Up to 60 mg
2	Up to 50 mg	Up to 80 mg
3	Up to 60 mg; increase by 5–10 mg up to every 3–5 days thereafter for opioid withdrawal/cravings	Up to 100 mg; increase by 10–20 mg up to every 3 days thereafter for opioid withdrawal/cravings

The study was reviewed by the University of California, San Francisco Institutional Review Board #22–36527 and deemed exempt.

### Data Sources and Abstraction

We used structured and unstructured EHR data to conduct our evaluation. Two members of the study team (MM and TB) abstracted EHR data. This included demographic, clinical, and hospitalization characteristics, including age, gender identity, race/ethnicity, housing status, length of stay, discharge diagnosis, discharge disposition, substance use disorders, past medication for OUD, daily hospital methadone doses, and additional FAO doses in morphine milliequivalents (MMEs). We also abstracted race/ethnicity and gender identity for patients who were evaluated for RMT but did not receive it to ensure the RMT was being implemented equitably.

To identify adverse events among patients who received the RMT, we searched the chart and reviewed notes and medication administration for sedation, falls, naloxone administration, intensive care unit transfer, intubation, and death. For all patients who experienced an adverse event, we additionally reviewed these for additional sedating medications, acute kidney injury, day of occurrence of adverse event within hospitalization, and the subsequent clinician intervention. Clinicians rated adverse events using the Common Terminology Criteria for Adverse Events (CTCAE).^16^ CTCAE grade 1 adverse events are asymptomatic or mild and do not require intervention; grade 2 are moderate and may require minimal noninvasive intervention; grade 3 are severe but not life-threatening and require hospitalization or prolongation of hospitalization; grade 4 are life-threatening and require urgent intervention; and grade 5 result in death.^16^ The author group met to obtain consensus by majority on CTCAE grading, etiology, outcome, and likelihood of being related to RMT (unrelated, possibly, probably, or definitely related).

### Outcomes

Our primary outcome for this case series was adverse events. Secondary outcomes included median time to 100 mg of methadone, FAO doses by day, and self-directed discharge.

## RESULTS

**Figure 1:**
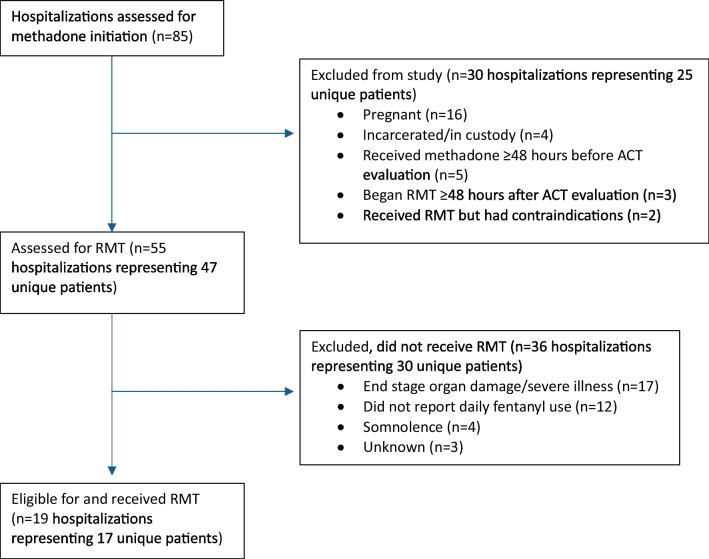
Consort diagram of hospitalizations assessed for new methadone initiation and those that received rapid methadone titration (RMT).

During the study period, ACT evaluated 85 hospitalizations for new methadone initiation (Fig. 1), and 55 met the study inclusion criteria and were assessed for RMT. Of these, 19 (34.5%) hospitalizations representing 17 unique patients were eligible, and received RMT. One patient received methadone via RMT on three separate hospitalizations, with more than 3 weeks between discharge and admission.

Patients who received methadone via the RMT protocol had a median age of 40.0 years (interquartile range [IQR] 34.0–43.0), with the majority being male (88.2%), White (82.4%), and unhoused (76.5%) (Table 2). Most had a concurrent substance use disorder, with the most prominent being stimulant use disorder (76.5%); most had self-reported prior experience with methadone (82.4%), and all had prior self-reported experience with methadone or buprenorphine. Most were hospitalized due to skin and soft tissue or complex infections (94.7%). The median length of stay was 6.0 (IQR 4.5–11.5) days, and 1 (5.3%) had a self-directed discharge.

**Table 2:** Sociodemographics and Clinical Characteristics of Patients Who Received RMT (*n* = 17)

	Received RMT (*n* = 17)
Race/ethnicity, *n* (%)	
Black or African American	1 (5.9)
Latine	0 (0.0)
White	14 (82.4)
Other	2 (11.8)
Gender identity*, *n* (%)	
Female	2 (11.8)
Male	15 (88.2)
Age (years), median (IQR)	40.0 (34.0–43.0)
Housing status, *n* (%)	
Housed	4 (23.5)
Unhoused	13 (76.5)
Concurrent SUD, *n* (%)**	
Alcohol	3 (17.6)
Benzodiazepine	3 (17.6)
Tobacco	11 (64.7)
Stimulants	13 (76.5)
History of past medication for addiction treatment, *n* (%)**	
Both	10 (58.8)
Buprenorphine	13 (76.5)
Methadone	14 (82.4)
Neither	0 (0.0)

^*^All patients identified as cisgender

^**^Not mutually exclusive

We reviewed exclusion criteria by race/ethnicity and gender identity among the 36 hospitalizations representing 30 patients who were assessed for RMT but did not receive it (Fig. 1). We found that Black individuals were largely excluded for end-stage organ damage or critical illness and non-daily fentanyl use (data not shown). Latine, White, and other individuals were excluded for end-stage organ damage or critical illness, and female patients for non-daily fentanyl use. Three patients (one Latine, one Black, and one White) were excluded for unknown reasons.

### Adverse Events

Four of 19 (21.2%) who received RMT had a chart documented concern for sedation. All were mild or moderate grade by the CTCAE (Table 3). Two resulted in decreasing the methadone dose by 10 mg, though all four required further uptitration. All had a co-occurring methamphetamine use disorder and were getting additional FAO (including one who was receiving scheduled FAO), and two were receiving additional sedating medications. Three events were thought to be possibly RMT-related, and one was thought to be probably RMT-related.

**Table 3:** Characterization of Adverse Events, Responses, and Likelihood of Each Resulting from RMT Among Hospitalizations Who Received RMT (*n* = 19)

**#**	Type	Day of methadone dose	Methadone dose during event (mg)	Methadone dose at discharge (mg)	Grade*	Response	Concurrent substance use disorders	Other sedating medications/factors	Likelihood related to RMT**
1	Sedation	2	60.0	100.0	1-Mild	None	Methamphetamine, tobacco	Hydromorphone, oxycodone	Possibly
2	Sedation	2	40.0	140.0	2-Moderate	Methadone dose maintained for a day	Methamphetamine, tobacco	Hydromorphone, oxycodone	Possibly
3	Sedation	6	80.0	120.0	2-Moderate	Methadone dose decreased by 10 mg	Methamphetamine, benzodiazepines	Gabapentin, hydromorphone, acute kidney injury	Probably
4	Sedation	14	120.0	120.0	2-Moderate	Methadone dose decreased by 10 mg	Alcohol, methamphetamine	Gabapentin, hydromorphone, olanzapine, oxycodone	Possibly

^*^Grades defined according to Common Terminology Criteria for Adverse Events (CTCAE): 1-Mild, 2-Moderate, 3-Severe, 4-Life threatening, and 5-Death

^**^Likelihood related to RMT determined to be either unrelated, possibly related, probably related, or definitely related

Additionally, two patients received RMT despite meeting exclusion criteria. Among these, one had end-stage renal disease and experienced sedation that was thought to be possibly RMT-related. There were no other documented adverse events among all patients who received the RMT protocol.

### Median Time to 100 mg of Methadone and FAO Dosing

Hospitalizations who received RMT per protocol achieved a median methadone dose of 100 mg by day 6. Table 4 includes the median daily methadone doses and additional FAO MME during hospital days 1–7. FAO doses peaked on day 5, while methadone doses increased throughout hospitalization.

**Table 4:** Median Daily Methadone Doses and Morphine Milliequivalents (MMEs) of Additional Full Agonist Opioids (FAO) Among Hospitalizations Who Receive the RMT (*n* = 19)

		Methadone		FAO	
Day*	Total N**	Number who received methadone	Median daily methadone dose mg (IQR)	Number who received FAO	Median FAO dose in MMEs (IQR)
1	19	19	40.0 (30.0–40.0)	18	90.0 (30.0–180.0)
2	19	19	60.0 (50.0–60.0)	19	108.0 (61.9–255.0)
3	18	18	80.0 (60.0–80.0)	17	121.3 (63.0–187.5)
4	18	18	80.0 (80.0–100.0)	17	116.6 (60.0–180.0)
5	12	12	90.0 (77.5–100.0)	12	144.0 (60.0–180.0)
6	11	11	100.0 (80.0–100.0)	10	90.0 (57.0–190.0)
7	8	8	100.0 (87.5–105.0)	8	67.5 (45.3–135.0)

^*^Day hospitalization first received methadone, not the day of hospitalization

^**^Number remaining hospitalized

*MMEs*, morphine milliequivalents in mg; *IQR*, interquartile range; *RMT*, rapid methadone titration

## DISCUSSION

We implemented a novel RMT protocol for hospitalized patients with OUD and daily fentanyl use initiating methadone and found that one in three assessed for RMT received it. Among those who did not receive RMT, most were appropriately excluded. Overall, our main findings were (1) a quarter of hospitalizations that received RMT experienced sedation that was mild-moderate range; (2) the median methadone dose was in the therapeutic range of 100 mg by day 6; and (3) self-directed discharge rates were low.

This case series adds to the existing literature on RMT during hospitalization. Hospitalization allows for close monitoring and evaluation of patients and multiple hospitals have leveraged this to offer RMT to patients with fentanyl use disorder.^17–21^ To our knowledge, only two other studies have reported outcomes from comparable RMT protocols, including one from our health network’s opioid treatment program in the outpatient setting, and an inpatient study from Portland, OR, allowing for doses up to 60 mg, 70 mg, 80 mg, and 100 mg on hospital days 1–4, respectively.^20,22^

Though our RMT protocol can achieve 100 mg by day 3, only two hospitalizations did, potentially due to multiple reasons. First, the RMT protocol could only be implemented with ACT evaluation and reevaluation at peak serum methadone effect daily before administering additional methadone to reach the maximum dose allotted per day. We were limited by in-person ACT availability and the day and time of the consult. Second, we included patients with concurrent benzodiazepine and alcohol use disorders, whom other inpatient studies have excluded. Withdrawal management and treatments for these, as well as stimulant use disorder withdrawal, may have caused sedation, limiting the ability to up-titrate methadone.^23,24^ Third, hospitalized patients may face critical illnesses and not tolerate methadone titrations as quickly as ambulatory patients with fentanyl use disorder.^22^ Further, patients receiving additional FAO during hospitalization, as our patients did, may not need to up-titrate methadone as quickly to alleviate withdrawal, compared to patients not receiving FAO. Additional reasons that may explain why patients did not achieve maximum daily methadone dosages available from RMT included patients not wanting to up-titrate methadone, and patients receiving other sedating medications (e.g., neuropathic agents, antipsychotics, sleep aids, benzodiazepines) that limited methadone uptitration.^25^ We did find that with the increasing daily dose of methadone (and methadone potentially reaching higher blood levels), median FAO MMEs peaked on day 5. It is possible that as the methadone dose increased, fewer FAO were required to treat ongoing withdrawal or that fewer FAO were required due to other reasons, such as improvement in pain.

Only one (5.3%) hospitalization that received RMT had a self-directed discharge. This is a low rate of self-directed discharge compared to prior local data showing rates between 11 and 24% and national data showing rates up to 44% among patients with substance use disorders.^5,14,25,26^ This may not be due to RMT, but by overall adequate opioid withdrawal management which may reduce the rate of self-directed discharges. Both RMT and FAO may have helped with and are consistent with other studies elucidating this trend as well as people with OUD sharing needing faster and higher doses of methadone in the setting of fentanyl.^9,25,27^

Notably, among those evaluated for the RMT protocol, we appropriately excluded almost two-thirds for clinical reasons, pointing to the need for careful patient selection. Four patients who were appropriately selected to receive RMT experienced oversedation, and one was thought to be probably RMT-related. This again points to the need for careful monitoring before increasing the methadone dose as the RMT protocol requires and was appropriately done by the ACT, who evaluated patients at peak methadone effect daily before recommending additional doses of methadone. However, even then, two required decreasing the methadone dose by 10 mg though all four required further uptitration of methadone. In addition, two patients who met RMT protocol exclusion received it. Of these, one had end-stage renal disease and experienced sedation that was possibly due to the RMT. This patient had difficulty tolerating hospitalization and had in-hospital substance use on multiple occasions. The patient had sedation documented during the first several weeks of hospitalization and had a complex course given a severe infection requiring multiple surgeries. The ACT may have proceeded with the RMT despite exclusion criteria due to the patient’s difficulty tolerating hospitalization. These findings emphasize the need to continuously review individual cases to ensure the appropriate application of new interventions in the hospital setting and identify areas for ongoing improvement.

There are several limitations to this study. First, it is a small case series based on retrospective chart review and reports observational data, limiting our ability to determine causality and generalizability. This study does not provide outcome and follow-up data to show impact (e.g., linkage, treatment retention) beyond hospitalization. Our hospital currently does not have a structured mechanism for documenting in-hospital substance use concerns, and this could have contributed to some of the oversedation we saw. We also included three hospitalizations from one patient in our sample, and our analysis does not adjust for the clustering of hospitalizations at the individual level. Finally, the RMT was initiated by the ACT, who carefully selected and evaluated patients, which may limit implementation among hospitals without an ACT.

Future studies comparing RMT with traditional methadone titrations should provide longer-term data that captures more hospitalizations that received RMT, evaluates safety, includes post-discharge outcomes, and presents qualitative data on how RMT affects the patient experience. We excluded pregnant patients from this evaluation and given faster methadone metabolism during pregnancy, it will be important to evaluate RMT outcomes among pregnant patients.^28^ Finally, it would be good to compare how outcomes would differ if RMT was implemented without additional FAO compared to RMT with additional FAO. The role of RMT will continue to be refined over time.

## CONCLUSION

In this small case series, with careful patient selection and ACT evaluation, we implemented a RMT protocol for hospitalizations with fentanyl use disorder initiating methadone who were not critically ill with few adverse events other than mild-moderate sedation, even among those receiving FAO and with concurrent substance use disorders. RMT may help alleviate opioid withdrawal and help patients complete hospitalization.

## Supplementary Information

Below is the link to the electronic supplementary material.

ESM 1 Supplementary file1 (DOCX 314 KB)
