# Supplementary material for: Early Results of Implementing Rapid Methadone Titration for Hospitalized Patients: A Case Series
Source: J Gen Intern Med. 2025 Jan 13;40(13):3104–10. doi: 10.1007/s11606-024-09341-1 (PMC12508316; doi:10.1007/s11606-024-09341-1)
Supplement: Supplementary file 1 — Supplementary file1 (DOCX 314 KB) [file 11606_2024_9341_MOESM1_ESM.pdf]

ZUCKERBERG SAN FRANCISCO GENERAL
Hospital and Trauma Center

ZSFG Addiction Care Team (ACT) Guideline: Rapid Methadone Titration for Hospitalized Patients

This guideline is intended for the initiation of methadone treatment for people with opioid use disorder (OUD), specifically those who have fentanyl use disorder with high opioid tolerance and who have the desire to initiate methadone treatment for OUD. This guideline should only be used upon ACT recommendation.

Please refer to the "ZSFG Inpatient Management of Opioid Use Disorder (OUD) Guideline: Methadone" for information related to traditional (non-rapid) methadone administration.

This guideline is intended to be used as guidance; however, there will be clinical scenarios where deviation from this guideline may be required (e.g, use of higher doses in pregnant patients, etc). Exceptions require appropriate communication between the Provider, RN, and PharmD.

Patient Selection

Patient selection is key. Patients will be carefully evaluated to ensure that they meet appropriate criteria to be initiated on this protocol. Prior to initiation, ACT will ensure all inclusion criteria have been met and all exclusion criteria have been reviewed. Additionally, ACT will review current inpatient and outpatient medications to assess for drug-drug interactions that may alter methadone metabolism as well as those with overlapping side effect profiles.

Inclusion Criteria	Exclusion Criteria
▪ Fentanyl use disorder<br>▪ High opioid tolerance as determined by ACT<br>▪ Urine toxicology is positive for the presence of fentanyl and it is established that this is not reflective of fentanyl administered at ZSFG, but rather a fentanyl use disorder<br>- Exception: pregnant patients. Refer to Urine Toxicology Testing in the Birth Center and Nursery Policy 65.0.	▪ Refer to contraindications described in the FDA package insert<br>▪ Age > 65 years<br>▪ Severe malnutrition<br>▪ Active ventricular arrhythmias<br>▪ Active acute coronary syndrome<br>▪ Disease processes w/ evidence of end-organ damage, including but not limited to:<br>- Moderate-severe lung disease (e.g. COPD, ILD)<br>- End stage renal disease<br>- Decompensated liver disease<br>- Decompensated heart failure<br>- Encephalopathy/cognitive impairment

EKG Monitoring

Obtain an EKG after a patient has received ≥100 mg of methadone daily for at least 4 consecutive days.

- If an EKG has been obtained prior to methadone 100 mg during the current admission, please review EKG for QTcF interval. In general, would like the QTcF interval to be < 500 ms. If the QTcF interval is >500 ms, ACT will provide guidance to the primary team regarding methadone titration and administration.

<table>
<tr><th colspan="4">Dosing and Monitoring Guideline</th></tr>
<tr><th>Day</th><th>Dosing</th><th>Daily Max Dose</th><th>Monitoring</th></tr>
<tr><td>1</td><td>▪ Assess sedation score. Administer methadone 40 mg PO x 1 if no concern for over-sedation.
▪ ACT/RN will assess patient at peak (4 hours after methadone dose) to evaluate for sedation.
▪ If patient continues to have withdrawal or cravings and is not over-sedated, ACT will instruct the primary team to order up to 20 mg more of methadone.
▪ The patient can also be supported with additional full opioid agonists & adjunct medications PRN for pain or w/d. See “Opioid Withdrawal PRN Medications” EPIC panel.</td><td>60 mg</td><td rowspan="5">▪ Place ALL patients on continuous pulse oximetry first the first 5 days.
▪ Of note, patients receiving other full opioid agonists or sedating medications (e.g. gabapentin, benzodiazepines) are at higher risk of sedation.
▪ RN to monitor sedation score before and 4 hours after each dose of methadone is administered. RN to monitor vital signs every 4 hours while methadone is being up-titrated (first 120 hours [5 days]).
▪ RN to notify primary team for:
- Respiratory rate ≤ 8
- O_2 saturation < 90%. For pregnant patients O_2 saturation < 94%.
- Ramsay score ≥ 4 (med-surg or L&D units) or RASS score of -3, -4, -5 (ICU)</td></tr>
<tr><td>2</td><td>▪ Assess sedation score. Administer methadone 60 mg PO x 1 in the morning (or total methadone received on day 1) if no concern for over-sedation.
▪ ACT/RN will assess patient at peak (4 hours after methadone dose) to evaluate for sedation.
▪ If patient continues to have withdrawal or cravings and is not over-sedated, ACT will instruct the primary team to order up to 20 mg more of methadone.
▪ The patient can also be supported with additional full opioid agonists & adjunct medications PRN for pain or w/d. See “Opioid Withdrawal PRN Medications” EPIC panel.</td><td>80 mg</td></tr>
<tr><td>3</td><td>▪ Assess sedation score. Administer methadone 80 mg PO x 1 in the morning (or total methadone received on day 2) if no concern for over-sedation.
▪ ACT/RN will assess patient at peak (4 hours after methadone dose) to evaluate for sedation.
▪ If patient continues to have withdrawal or cravings and is not over-sedated, ACT will instruct the primary team to order up to 20 mg more of methadone.
▪ The patient can also be supported with additional full opioid agonists & adjunct medications PRN for pain or w/d. See “Opioid Withdrawal PRN Medications” EPIC panel.</td><td>100 mg</td></tr>
<tr><td>4</td><td>▪ Assess sedation score. Administer methadone 100 mg PO x 1 in the morning (or total methadone received on day 3) if no concern for over-sedation.
▪ ACT/RN will assess patient at peak (4 hours after methadone dose) to evaluate for sedation.
▪ The patient can also be supported with additional full opioid agonists & adjunct medications PRN for pain or w/d. See “Opioid Withdrawal PRN Medications” EPIC panel.</td><td>100 mg</td></tr>
<tr><td>≥ 5</td><td>▪ Assess sedation score. Administer methadone 100 mg PO x 1 in the morning (or total methadone received on day 4). On day 5 and every third day thereafter, methadone dose may be increased by up to 20 mg after ACT evaluates the patient at peak (4 hours after methadone dose).
▪ Obtain EKG after 4 days of methadone ≥100 mg. Refer to “EKG monitoring” section for more information.</td><td>Variable based on titration</td></tr>
</table>

*** Deviation from these titration recommendations may occur per ACT discretion, especially in pregnant patients and those who are rapid metabolizers of methadone as these patients may reach steady state sooner.*

References

1. Klaire, S, Fairbairn N, Ryan A, et al. Safety and Efficacy of Rapid Methadone Titration for Opioid Use Disorder in an Inpatient Setting: A Retrospective Cohort Study. J Addict Med. 2023; 17(6): 711-713.
2. Racha S, Patel SM, Harfouch LTB, et al. Safety of rapid inpatient methadone initiation protocol: A retrospective cohort study. J Substance Use and Addiction Treatment. 2023: 148.
3. Buresh M, Nahvi S, Steiger S, Weinstein ZM. Adapting methadone inductions to the fentanyl era. J Subst Abuse Treat. 2022. Oct;141:108832. doi: 10.1016/j.jsat.2022.108832. Epub 2022 Jun 27. PMID: 35870437
4. Hemmons P, Bach P, Colizza K, Nolan S. Initiation and Rapid Titration of Methadone in an Acute Care Setting for the Treatment of Opioid Use Disorder: A Case Report. J Addict Med. 2019; 13(5):408-411.
5. Sarah Ickowicz & Mark McLean (2021): Case report: rapid inpatient methadone titration during pregnancy, Journal of Substance Use, DOI: 10.1080/14659891.2021.1953167.
